# Flap Impaling: A Simple and Effective Technique in Soft Tissue Reconstruction of Complex Extremity Wounds

**DOI:** 10.1055/s-0045-1807284

**Published:** 2025-10-29

**Authors:** Sathish Kumar Jayaraman, Abiramie Chellamuthu, Srinivasan Venugopal, Vishnu Priya Vasudevan

**Affiliations:** 1 Department of Plastic Surgery, Sri Ramachandra Institute of Higher Education and Research, Chennai, Tamil Nadu, India

**Keywords:** flap impaling, soft tissue reconstruction, compound defects, free flaps

## Abstract

Composite defects due to trauma present with compound comminuted fractures with soft tissue loss. Stabilization requires multiple Schanz pins across the fracture segments and flap cover for soft tissue reconstruction. These pins hinder flap inset and may require complex planning with multiple flaps. A simple approach is to impale the flaps over these pins to achieve flap inset without disturbing the skeletal fixation. This method of impaling was utilized for free as well as pedicled fasciocutaneous and muscle flaps. A stab incision is made and careful dissection is done to create a passage for the pins without injuring the vascularity of these flaps. In this manner multiple pins can be passed as needed. Flap inset is then completed. Slight modifications in threading the pins are required depending on whether a free or a pedicled flap is used. This study includes 16 flaps of which 15 were for lower limbs and 1 was for upper limb reconstruction. Pedicled flaps used were 13, the most common being inferiorly based fasciocutaneous flaps. Free flaps used were three, which included two muscle flaps and one fasciocutaneous flap. Two flaps were impaled in three places. All the flaps survived without any loss. Suture line dehiscence in four flaps was managed conservatively. Impaling the flaps on Schanz pins is a simple procedure to achieve soft tissue reconstruction in compound defects. Knowledge of the vascular anatomy and blood supply of the flaps is imperative in protecting the pedicle and maintaining the vascularity.

## Introduction

“Orthoplastic” approach is a term used for multidisciplinary modality of lower extremity reconstruction involving plastic and orthopaedic surgeons for optimal patient outcomes. Composite defects due to trauma require an organized approach utilizing the reconstructive ladder. Small defects can be covered with local flaps. Free tissue transfer is indicated in the repair of injuries that are highly complex, with involvement of large surface area. 1 Muscle flaps have direct vascular pedicles and fasciocutaneous flaps have perforators contributing to the blood supply. 2

Polytrauma patients who present to our emergency department are managed after initial debridement by skeletal stabilization with external fixators. Most of these patients undergo definitive skeletal fixation after soft tissue coverage. In such instances, the pins of external fixators may hinder flap inset and will require complex planning with multiple flaps. We have devised a simple approach where the flaps are impaled on the pins during the procedure of soft tissue cover. The definition of “impale” is to push a sharp pointed object through somebody/something. We present our experience of impalement with various flaps including pedicled and free flaps, with modifications depending on the vascularity of the flaps used.

## Materials and Methods

We present a case series of all the patients who underwent flap impaling during the initial soft tissue reconstruction at our institution over a 5-year period. These patients were either referred from other hospitals with external fixators or managed by our team with initial debridement and external fixation. Impaling is not indicated if the pins are in close proximity to the wound. In these cases, the edge of the flap can be split to accommodate the pin. Similarly, the fixation has to be uniaxial and uniplanar, for impalement of the flaps. The flaps utilized were pedicled as well as free fasciocutaneous and muscle flaps. Preoperatively, Doppler was used to identify the vessels/perforators and marking their course on the flaps. Flap elevation was performed after planning in reverse. In the pedicled flaps, we also visually see the vessels if visible or digitally palpate the vessels before impaling.

**Fig. 1:**
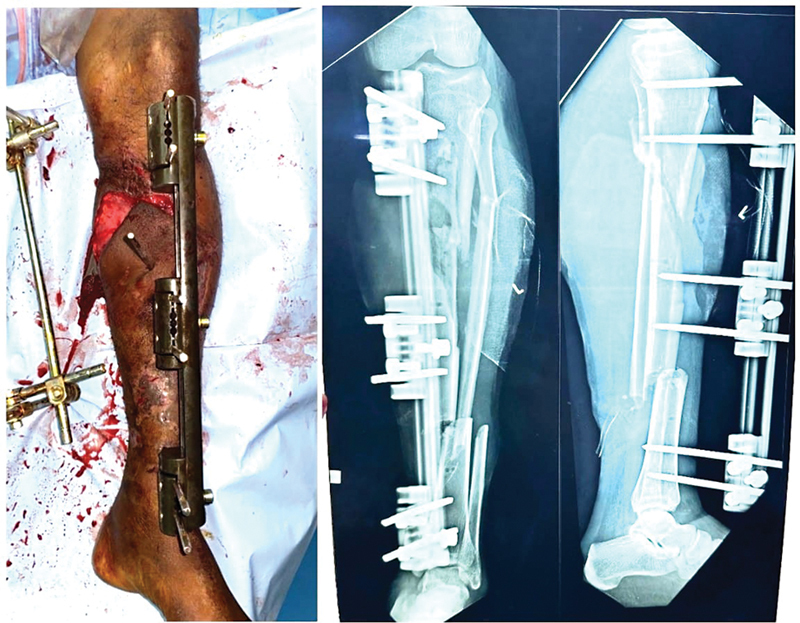
Impaling of fasciocutaneous flap.

For pedicled flaps, the horizontal construct is retained and reduction is maintained, only the external fixator pins that hinder the inset are withdrawn with the help of the orthopaedic team. Flap is then brought in to the position of future inset and new pin position is then marked on the flap. Pedicle vessels are safeguarded while impaling the flap. The stab incision required for pin placement is made and careful dissection is performed from skin through all the layers of the flap. The pin is then passed through the incision, drill is used to drive the pin through the bone to restore the fixation ( Figs. 1 2 3 ). Multiple pins can be passed in this manner if required. Flap inset is then completed.

**Fig. 2:**
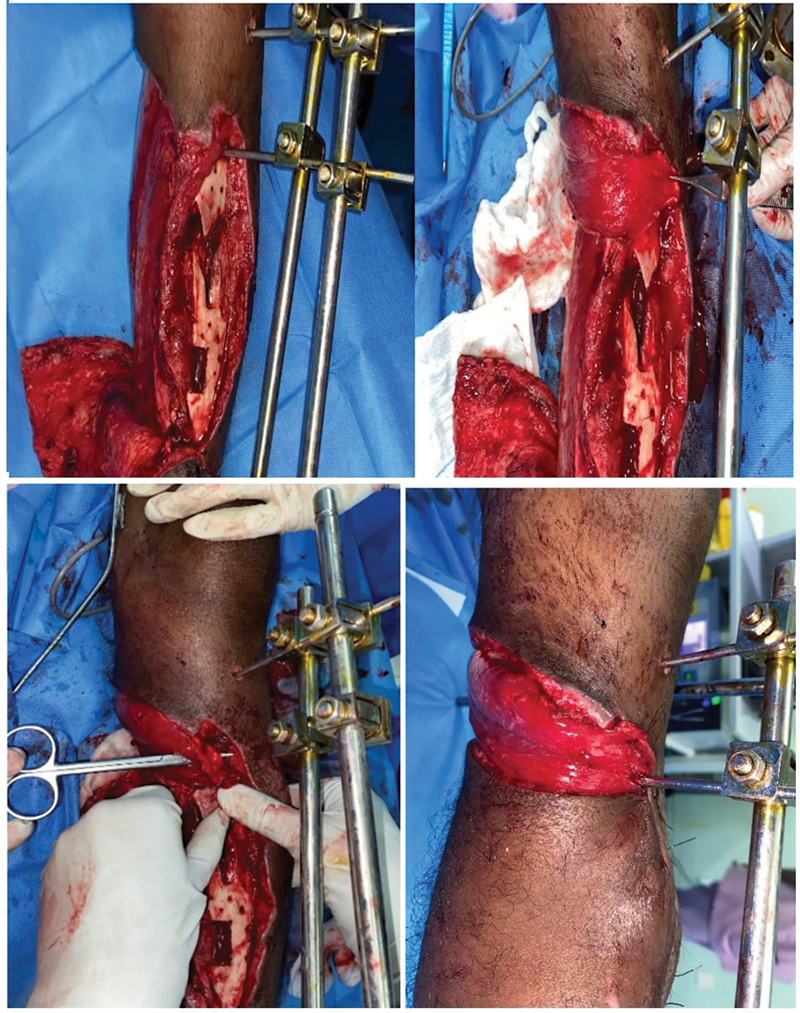
Impaling of medial gastrocnemius muscle flap.

**Fig. 3:**
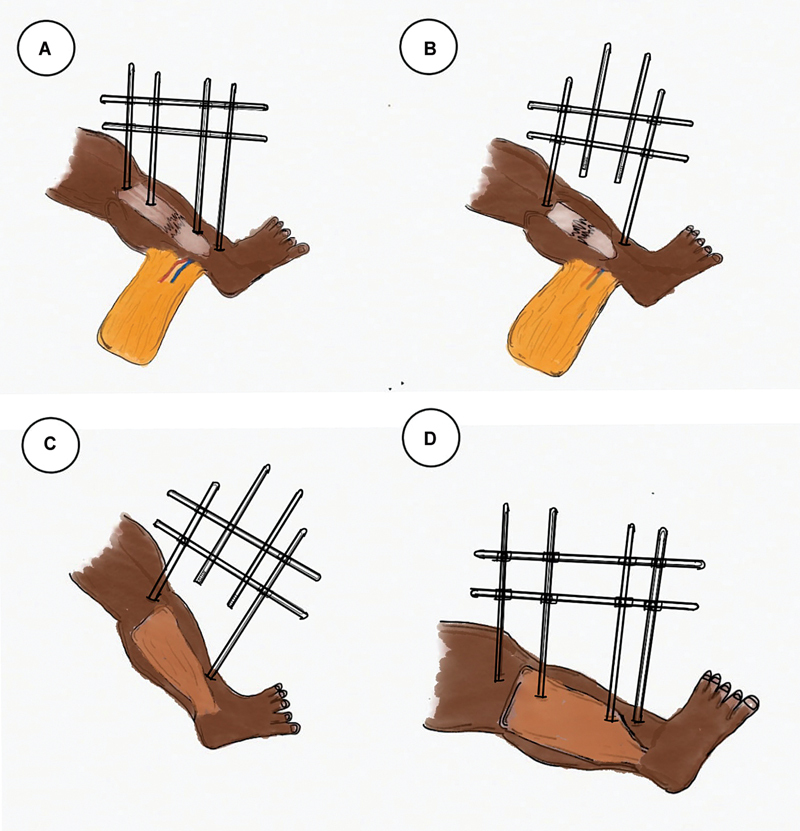
( **A** ) Inferiorly based fasciocutaneous flap elevated. ( **B** ) The Schanz pins which come in the way of inset removed. ( **C** ) Flap positioned to inset. ( **D** ) After impaling the pins over the flap.

**Fig. 4:**
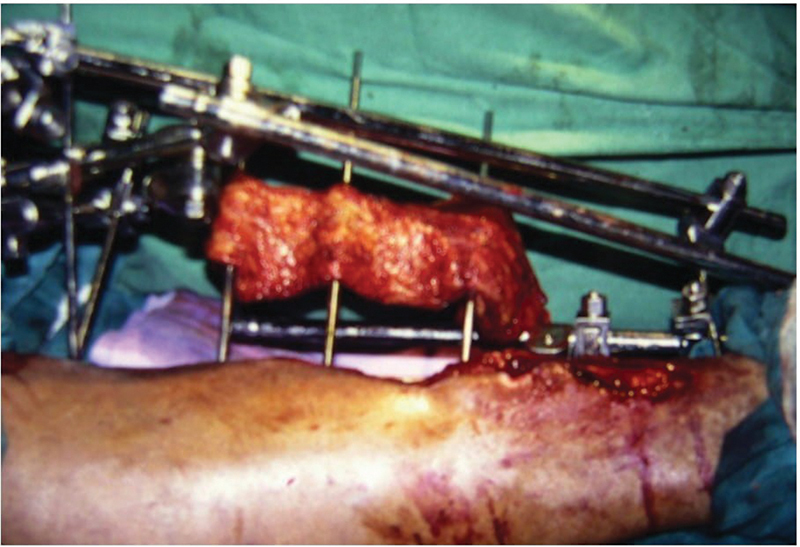
Impaling the rectus abdominis muscle flap.

**Fig. 5:**
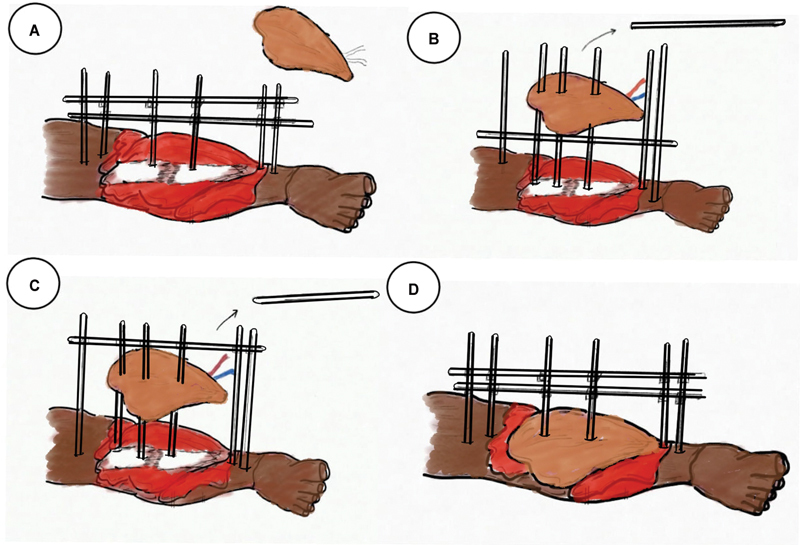
( **A** ) Free muscle flap. ( **B** ) Outer connecting rod removed and the flap impaled over the inner rod. ( **C** ) Outer rod is reapplied and inner rod is removed maintaining the fracture stability. ( **D** ) Flap slid down to the defect, the inner rod is placed back and anastomosis done.

The impalement of a free flap is achieved with the following steps. External fixators should have two horizontal rods connecting the pins for stable fixation. The outer rod is removed, the flap is impaled after careful dissection avoiding the pedicle, and the outer rod is placed back. Flap is now between the inner and outer horizontal rod. The inner rod is then removed, the flap is slid down, and the inner rod is placed back. In this manner, the reduction is not disturbed and fracture readjustments leading to flap shearing and flap loss are avoided ( Figs. 4 and 5 ). Flap inset is completed and the anastomosis is done.

All these flaps were followed up in the postoperative period for a minimum period of 6 months. Data are collected for flap survival, necrosis, dehiscence, and wound infection.

## Results

The study included 16 flaps of which 15 were for lower extremity and 1 was done for upper extremity. Of the 15 flaps used for lower extremity, 10 were fasciocutaneous flaps and 5 were muscle flaps. Pedicled flaps were 13, with inferiorly based fasciocutaneous flaps being the most common, followed by medial gastrocnemius muscle flaps. Free flaps used were three, which included one latissimus dorsi, one rectus abdominis muscle flap, and one anterolateral thigh flap.

All the defects were caused by trauma due to road traffic accidents. Regarding the location of the defects, five were in the middle third of the leg, three were in the distal third leg, and seven were large defects involving the middle and distal third region, and one was in the arm.

**Fig. 6:**
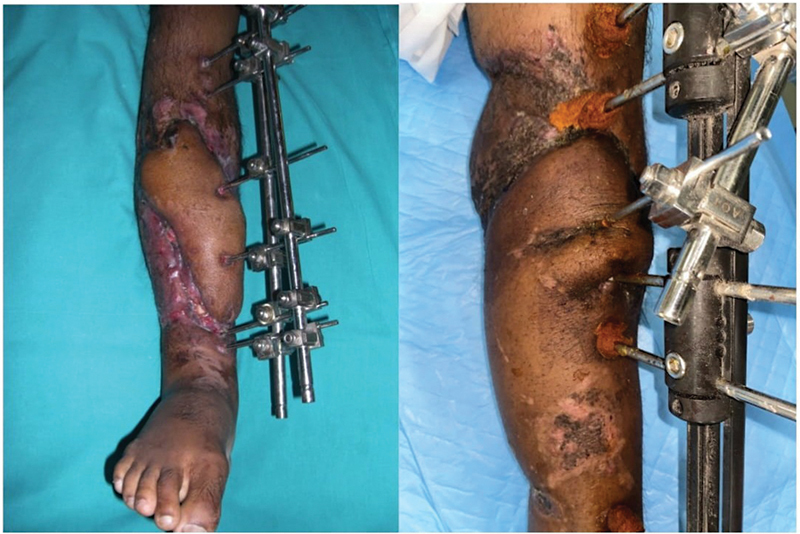
Free anterolateral thigh flap and pedicled inferiorly based fasciocutaneous flap.

All the flaps survived without any necrosis ( Fig. 6 ). The most common complication was a postoperative suture line infection seen in four pedicled fasciocutaneous flaps. Infections were managed conservatively with appropriate antibiotics. Patients were followed up for an average of 18 months. Two of the pedicled fasciocutaneous flaps required reelevation for further orthopaedic procedures.

## Discussion

High-velocity injuries often lead to fragmented tibia fractures, creating large defects with varying degree of vascular damage. 3 Since these wounds are heavily contaminated and have comminuted compound fractures with floating segments, initial orthopaedic stabilization is commonly done with external fixators. Though intramedullary nailing is the definitive fixation, the use of external fixators is considered a viable surgical option by many authors. 4 5 6 Three distinct types of fixators commonly used are wire and ring fixator, half-pin fixators, and hybrid fixators. The initial fixation should be rigid enough to reduce motion at the fracture sites. The diameter of the pin should be less than one-third of the bone diameter to prevent fracture. Noncomminuted fractures require at least two pins for each fragment. Two-plane fixation is preferred in comminuted fractures. The multiple segmental fragments can be attached with one or two pins and these can provide a “joystick manipulation” until adequate reduction is achieved. 4 Advantages of the external fixator system is the ease of application, simple additional fracture reduction if needed, and less bulky providing easy access to further plastic procedures. Wherever possible or feasible, plaster of Paris slabs can be used in addition to external fixators during flap reconstruction. Disadvantages include nonunion, pin site complications, and pin loosening. If the pin is removed, reinsertion of the new pin is done at a different site for stability.

**Fig. 7:**
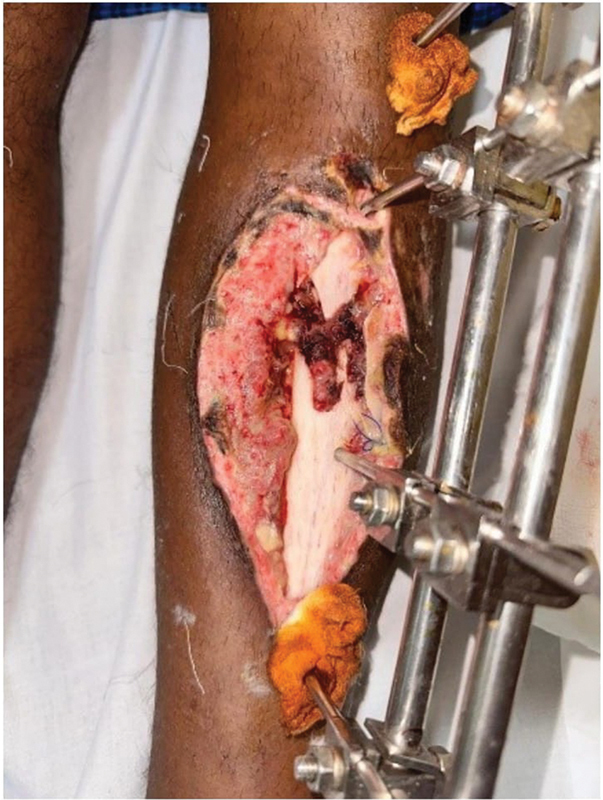
External fixator pins on bare bone.

In these scenarios of multisegment fractures with separate pins holding each fragment, bringing a flap between these pins is challenging ( Fig. 7 ). The flap planning for these defects should either have multiple extensions or single flap with multiple slits to reach between these pins, which may compromise the vascularity of the smaller extensions. A simpler approach is to impale the pins on the flaps with careful dissection avoiding the pedicles.

Cadaveric studies have shown definitive blood supply for the fasciocutaneous flaps. 7 Various anatomical studies have been done to clearly map out the perforators in the leg. Multiple posterior tibial perforators of clinical significance (diameter > 0.5 mm) were located at a mean distance of 6.64 cm from the medial malleolus. There was an average of 4.17 cutaneous perforating branches 8 per leg from the peroneal artery 9 and around four to five perforators from the anterior tibial artery. 2 7 Perforasomes from these perforating vessels are longitudinally connected by indirect linking vessels. They also get recurrent flow through the subdermal plexus. 10 These linking vessels are almost identical to the choke vessels reported by Taylor. 11

Since fasciocutaneous flaps receive blood supply from the perforators, which arborize with multiple linking vessels, it is safe to make a stab incision and impale these flaps well away from the pedicle.

Muscle flaps commonly used for reconstruction are elevated based on a dominant vascular supply and the course of these vessels has been documented by cadaveric studies. Rowsell et al have shown that the thoracodorsal artery immediately divides in to two muscle branches on reaching the deep surface of latissimus dorsi muscle. Multiple secondary branches arise from these and run through the substance of muscle from deep to superficial. 12 13 Similar documentations of the pedicle anatomies within the muscles have been published. 13 14 15 16 It is imperative to have this knowledge of vascular arborizations within the muscle before impaling the muscle to protect the pedicle.

Hence, both fasciocutaneous and muscle flaps can be safely impaled on the Schanz pin to achieve soft tissue reconstructions in complex defects. It is extremely important to elevate a flap of adequate size, taking care of the pedicle and providing tension-free inset. Impaling provides an additional benefit of strengthening the inset, especially if the flap goes over the convexity of the tibia. Most of the polytrauma coming to us are managed by staged approach wherein the reconstruction and soft tissue equilibrium is achieved first and then the definitive skeletal stabilization is done later if necessary. The cases with multiple segmental fractures will require at least one Schanz pin across each segment to stabilize the fractures. In these instances, flap impaling helps us achieve a soft tissue reconstruction without endangering fixation.

## Conclusion

Impaling the flaps on Schanz pins is a simple and effective way of achieving flap inset and soft tissue reconstruction without compromising the vascularity and fixation. Multiple pins can be impaled on the flaps safeguarding the pedicles. Critical knowledge of the vascular anatomy and blood supply of the flaps is imperative in preventing pedicle injury.
